# Repair or Degrade: the Thermodynamic Dilemma of Cellular Protein Quality-Control

**DOI:** 10.3389/fmolb.2021.768888

**Published:** 2021-10-27

**Authors:** Bruno Fauvet, Mathieu E. Rebeaud, Satyam Tiwari, Paolo De Los Rios, Pierre Goloubinoff

**Affiliations:** ^1^ Institute of Physics, School of Basic Sciences, École Polytechnique Fédérale de Lausanne—EPFL, Lausanne, Switzerland; ^2^ Department of Plant Molecular Biology, Faculty of Biology and Medicine, University of Lausanne, Lausanne, Switzerland; ^3^ Institute of Bioengineering, School of Life Sciences, École Polytechnique Fédérale de Lausanne—EPFL, Lausanne, Switzerland

**Keywords:** proteostasis, thermodynamics, protein repair, chaperones, protein degradation

## Abstract

Life is a non-equilibrium phenomenon. Owing to their high free energy content, the macromolecules of life tend to spontaneously react with ambient oxygen and water and turn into more stable inorganic molecules. A similar thermodynamic picture applies to the complex shapes of proteins: While a polypeptide is emerging unfolded from the ribosome, it may spontaneously acquire secondary structures and collapse into its functional native conformation. The spontaneity of this process is evidence that the free energy of the unstructured state is higher than that of the structured native state. Yet, under stress or because of mutations, complex polypeptides may fail to reach their native conformation and form instead thermodynamically stable aggregates devoid of biological activity. Cells have evolved molecular chaperones to actively counteract the misfolding of stress-labile proteins dictated by equilibrium thermodynamics. HSP60, HSP70 and HSP100 can inject energy from ATP hydrolysis into the forceful unfolding of stable misfolded structures in proteins and convert them into unstable intermediates that can collapse into the native state, even under conditions inauspicious for that state. Aggregates and misfolded proteins may also be forcefully unfolded and degraded by chaperone-gated endo-cellular proteases, and in eukaryotes also by chaperone-mediated autophagy, paving the way for their replacement by new, unaltered functional proteins. The greater energy cost of degrading and replacing a polypeptide, with respect to the cost of its chaperone-mediated repair represents a thermodynamic dilemma: some easily repairable proteins are better to be processed by chaperones, while it can be wasteful to uselessly try recover overly compromised molecules, which should instead be degraded and replaced. Evolution has solved this conundrum by creating a host of unfolding chaperones and degradation machines and by tuning their cellular amounts and activity rates.

## Introduction

### Protein Folding, Misfolding and Aggregation

**FIGURE 1:**
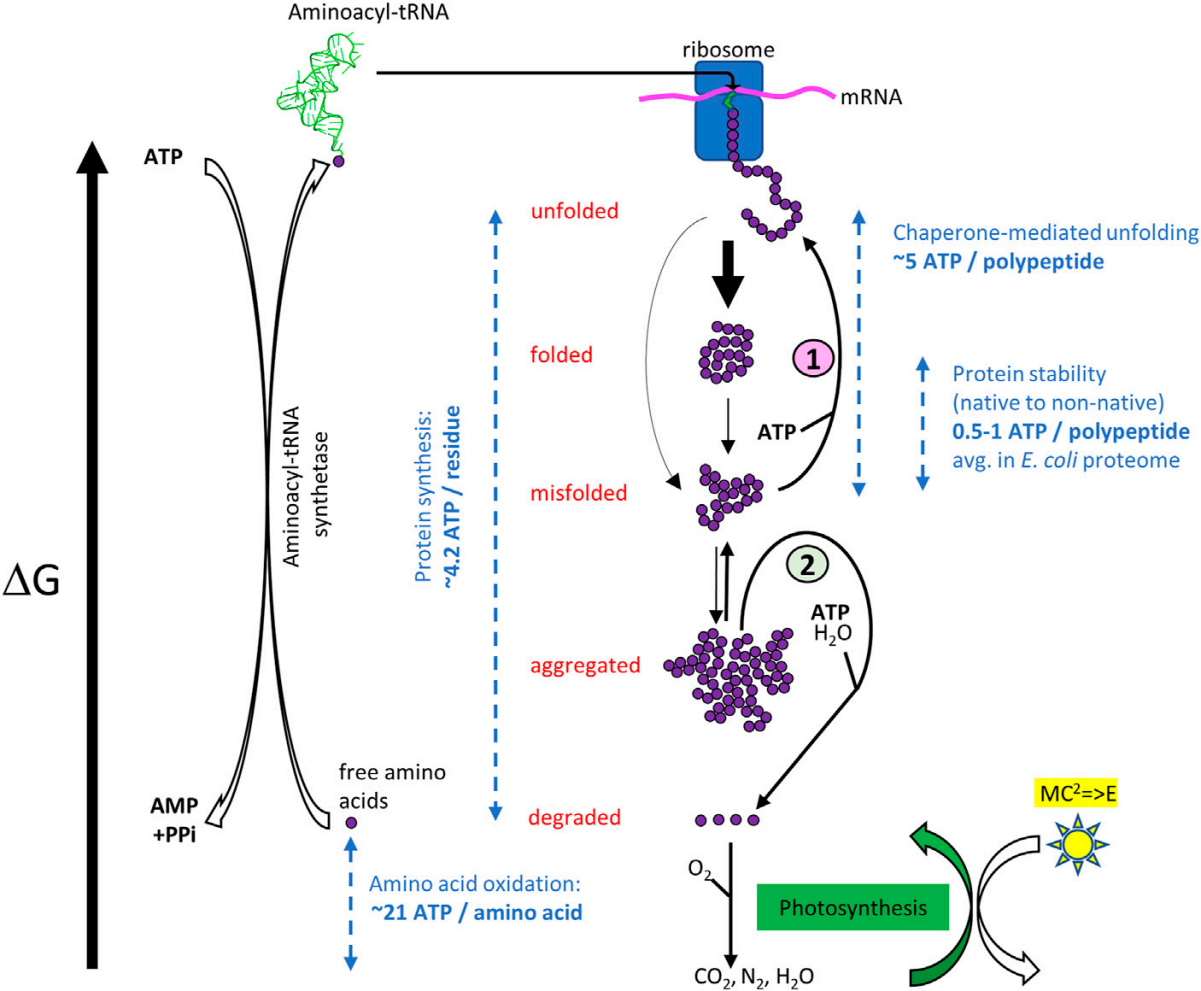
ATP-cost of the open, non-equilibrium free energy cycle of cellular protein homeostasis. Top. When forming in the ribosome, a nascent polypeptide is unfolded, and it has the highest free energy. It may then spontaneously collapse into the native state (folded) or reach metastable misfolded states (misfolded). Heat stress can reduce the kinetic barriers between states. Even without heat stress, the native state can partially unfold and convert into a misfolded state, which in turn converts into stable non-native oligomers (aggregated). (1) The misfolded and aggregated species can be structurally “repaired” into native species again by ATP-fueled unfolding chaperones, or (2) be degraded by proteases gated by ATP-fueled unfolding chaperones (Rothman and Kornberg, 1986). Free amino acids may further degrade into more stable inorganic molecules from which new amino acids can be synthetized, using energy initially originating from the sun, in the form of ATP generated by photosynthesis. ATP-consuming aminoacyl-tRNA synthetases may then load free amino acids with the highest free energy state, which in the ribosomes can spontaneously form less energetic peptide bonds into an unfolded polymer with a lower free energy. Dashed blue arrows indicate typical energy differences between various states, expressed as ATP equivalents (Jungas et al., 1992; Ghosh and Dill, 2010; Sharma et al., 2010; Kaleta et al., 2013).

On a free energy landscape, an ensemble of amino acids can be found in different energy states. As testified by the spontaneous hydrolysis of polypeptides in the presence of trypsin, polymerized amino acids are higher in free energy than when unpolymerized. Moreover, a single polypeptide chain can be found in different structural states: unfolded, which is in most cases inactive, natively-folded, which is generally biologically active, misfolded, which is inactive and often toxic. Misfolded species may oligomerize into amorphous aggregates and further evolve into increasingly stable and more compact fibrils (Figure 1). The unfolded state, such as when a nascent polypeptide exits from the ribosome, has the highest free energy and, as initially shown by Anfinsen et al., (Anfinsen, 1973), can spontaneously collapse into a specific native state without requiring assistance from other molecules. Noticeably, this is not necessarily the most stable state and when kinetic barriers are reduced by mutations or higher temperatures, it may spontaneously undergo transient unfolding and readily collapse into various misfolded states devoid of specific dedicated biological functions, which can be more stable. Misfolded species expose more hydrophobic surfaces to water (Natalello et al., 2013; Sharma et al., 2011) and at high concentrations, tend to further assemble into larger and more compact aggregates that may be more stable than the native state (Hartl and Hayer-Hartl, 2009) (Figure 1). By virtue of their exposed hydrophobic surfaces, misfolded polypeptides and aggregates may seek to interact with lipids and affect membrane permeability and activity (Lashuel and Lansbury, 2006; Mahul-Mellier et al., 2015). In metazoans, various aggregates thus cause inflammation and cell death, leading to degenerative diseases and aging (Goloubinoff, 2016). Whereas small aggregates and misfolded polypeptides may be soluble (Diamant et al., 2000), aggregates with many polypeptide chains are often more compact and less soluble. Sustained stress, heat shock in particular, may favor the formation of increasingly larger and more stable aggregates resisting artificial solubilization by urea, or by ATP-fueled disaggregating chaperones, as normally occurring in the cell (Figure 1). Because *de novo* synthetized polypeptides exit from the ribosome mostly unfolded, sequentially from the N- to the C-terminal end, they are given an optimal chance to orderly fold, first the N-terminal domains, then the C-terminal domains, leading to proper folding to the native state. In contrast, when a native protein is under heat-stress, misfolding N- and C-terminal domains may concomitantly occur, and improper distal interactions may take place to form stable aggregates.

In the wild, protein misfolding and aggregation may not necessarily be a severe problem for cells living mostly in a quiescent state, as in the case of terminally differentiated adult mammalian cells or of bacteria and archaea living on extremely limited sources of nutrients and energy, for example in the ocean abyss or deep in rocks (Hoshino and Inagaki, 2019; Merino et al., 2019). Under such extremely limiting conditions for growth, most ribosomes are expected to be unoccupied, the few polypeptides to be synthetized being only in replacement of those naturally damaged and degraded. In contrast, when exponentially growing microorganisms are artificially studied in rich media and saturating oxygen, as typically in a laboratory, their protein synthesis and quality control machineries are being maximally challenged and more errors are expected to occur on their folding pathway. Moreover, stresses, such as heat shock, may cause misfolding and aggregation of particularly labile proteins (Mogk et al., 1999). It is thus not fortuitous that point mutations in the bacterial chaperone GroEL, GroES, DnaK, DnaJ and GrpE had been initially identified in a screen of *E. coli* mutants that failed to massively synthetize and properly assemble T4 phage proteins at 37°C, also turned out to be affected in their growth above physiological temperature (Liberek et al., 1988; Wild et al., 1992; Georgopoulos and Welch, 1993).

### Evolution of Protein Repair and Degradation Machineries

It has been inferred that more than 3.5 billion years ago, the common ancestor to bacteria and archaea (LUCA) possessed a simple genome that encoded for relatively uncomplicated short proteins, generally uninclined to misfold and aggregate. LUCA likely used only two chaperones, HSP20 that can prevent protein aggregation, and Hsp60 that can use ATP hydrolysis to unfold and repair structurally-damaged small proteins. LUCA likely possessed a single chaperone-gated protease that can use ATP hydrolysis to unfold and degrade proteins that became irreversibly damaged (Rothman and Kornberg, 1986; Rebeaud et al., 2021). The evolutionary history of the sequential buildup of various families of chaperones and proteases can be tentatively evaluated by addressing which ones are currently encoded in the genomes of the simplest and the most complex free-living archaea and bacteria (Rebeaud et al., 2021) (Table 1). The genome of the very simple free-living TACK archaeon *Thermogladius calderae* (Mardanov et al., 2012), with only 1,414 genes, encodes for a single Hsp60 chaperone and a single PAN-20S protease (Horwitz et al., 2007). The genome of the very simple free-living Aquificae bacterium *Desulfurobacterium thermolithotrophum* (Göker et al., 2011) with only 1,496 genes, encodes for two chaperones, Hsp20 and Hsp60 and five proteases: ClpAP, ClpXP (Gottesman et al., 1998; Glynn et al., 2009; Zeiler et al., 2013), Lon (Thomas-Wohlever and Lee, 2002), FtsH (Bieniossek et al., 2006) and HslUV (Yoo et al., 1996). By contrast, the genome of the complex ASGARD *Heimdallarchaeota* archaeon (strain LC_2) (Zaremba-Niedzwiedzka et al., 2017), with 4,485 genes and of the complex Gammaproteobacteria *Escherichia coli* (Blattner et al., 1997) with 4,391 genes, both encode for five conserved chaperone families: Hsp20, Hsp60, Hsp70, Hsp90, and Hsp100. In contrast, the picture of the endo-cellular proteases did not evolve much further: a single PAN-20S remained in the most complex archaeon (with Lon in some ASGARDs), and the same complex network of five endo-cellular protease families (ClpAP, ClpXP, Lon, FtsH, HslUV) that were already present in the simplest bacteria, remained present in the most complex ones. This is suggesting that in the evolution of the first prokaryotes, the “protein degradation toolbox” of microorganisms became fully deployed earlier than a more complex and more versatile “protein repair toolbox,” which would have evolved later, hand-in-hand with the proteome complexification. Interestingly, *D. thermolithotrophum* harbors a full set of five different endo-cellular proteases, while lacking HSP70, the central hub of the chaperone network, as well as Hsp90 and ClpB. Therefore, in contrast to ClpB (Hsp100), whose disaggregase activity strictly depends on Hsp70, the endo-cellular proteases are unlikely to depend on HSP70 for their activity. This is particularly the case of ClpA (which is closely related to ClpB), but also the more distantly related AAA+ proteases.

**TABLE 1:** Absence (NO) or presence (YES) of individual core-chaperones and AAA+ proteases in the genomes of present-day free-living simple Archaea (TACK), simple Bacteria (Aquificae), more complex Archaea (Asgard) and more complex bacteria (Proteobacteria).

Domain	Archaea		Bacteria	
Clade	TACK	ASGARD	Aquificae	Proteobacteria
Organism	Thermogladius Calderae DSM 22663	Heimdallarchaeota archaeon (strain LC_2)	Desulfurobacterium thermolithotrophum DSM 11699	*Escherichia coli* K12
References	Mardanov et al. (2012)	Zaremba-Niedzwiedzka et al. (2017); Rebeaud et al. (2021)	Göker et al. (2011); Rebeaud et al. (2021)	Blattner et al. (1997); Rebeaud et al. (2021)
**Chaperones**				
HSP90	NO	YES	NO	YES
HSP100/ClpB	NO	YES	NO	YES
Hsp70	NO	YES	NO	YES
HSP20	NO	YES	YES	YES
HSP60	YES	YES	YES	YES
**Proteases**				
HslUV	NO	NO	YES	YES
FtsH	NO	NO	YES	YES
ClpAP	NO	NO	YES	YES
ClpXP	NO	NO	YES	YES
Lon	NO	YES	YES	YES
PAN-20S	YES	YES	NO	NO

### Principal Chaperone Components of the Proteostasis Network

**FIGURE 2:**
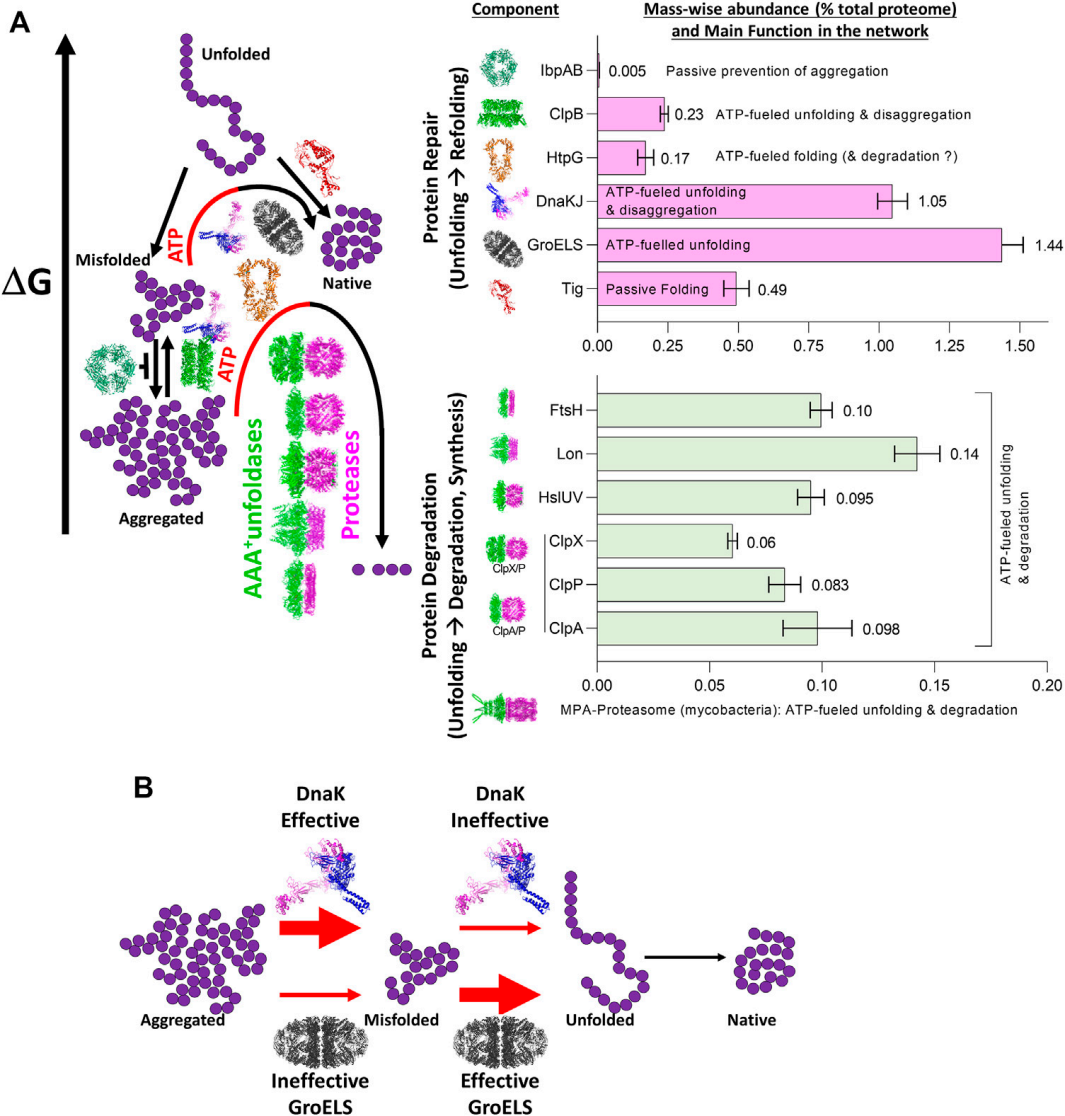
The role of the main bacterial chaperones and proteases. **(A)** Left panel: The main folding states of a polypeptide arranged by free energy, as shown in Figure 1. Whereas trigger factor (Tig) passively drives the native folding of unfolded polypeptides exiting the ribosome and HSP20 passively prevents misfolding and aggregation, GroEL, DnaK and ClpB can inject energy from ATP hydrolysis into the expansion of collapsed misfolded and aggregates species. HtpG may use ATP hydrolysis to complete the native folding of slow-folding intermediates from DnaK-DnaJ and failing that, may promote their degradation by proteases (Honoré et al., 2017; Fauvet et al., 2021). The bacterial proteases are gated by N-terminal chaperone-like AAA+ rings (green) that can unfold stable aggregates and thread them into a protease central cylinder (magenta), to be specifically degraded. Right panel: mass-wise abundances of chaperones and proteases from the protein homeostasis network; data from (Fauvet et al., 2021) **(B)** Example of synergism between bacterial HSP70 (DnaK) being effective (red thick arrow) at dismantling aggregates into individual misfolded polypeptides, but ineffective (red thin arrow) at unfolding individual misfolded polypeptides into natively refoldable species, and the bacterial Hsp60 system (GroELS) being ineffective at dismantling aggregates into individual misfolded polypeptides but being highly effective at unfolding individual misfolded polypeptides into natively refoldable species (thick black arrow).

The ATP-consuming chaperones and endo-cellular proteases involved in protein quality control share a common ability to gate their catalytic activities to substrates possessing the required physical characteristics: misfolded proteins in the case of chaperones, and either misfolded or tagged proteins in the case of proteases. Moreover, both need energy from ATP hydrolysis for their function: chaperones to actively unfold and thereby spontaneously *repair* the structure of misfolded substrates into native structure, and proteases to actively unfold and thereby spontaneously degrade them into peptides. Peptidases will in turn spontaneously degrade peptides into free amino acids, to be used to replace the damaged proteins (*degrade-to-replace*) (Figure 1). As mentioned, in *E. coli*, the protein repair part of the proteostasis network is mostly composed of the conserved chaperone families Hsp70s (DnaK, HscA), Hsp60s (GroEL), Hsp90s (HtpG), Hsp100 (ClpB) and Hsp20s (IbpA/B) (bacterial names in brackets), with their main co-chaperones (DnaJ, CbpA, DjlA, HscB, GrpE, GroES) (Finka and Goloubinoff, 2013). Indicating the importance of this chaperone network, its members contribute ∼3.3% of the total proteome mass of unstressed *E. coli* cells, compared to ∼1% for the endo-cellular proteases, with ∼0.6% of the total proteome belonging to quality-control proteases (Figure 2A), (Fauvet et al., 2021).

The three distinct chaperone families Hsp60, Hsp70 and Hsp100, which are composed of completely different constituting protomers also greatly differ structurally: Hsp60s are tetra- and hexa-decamers, the Hsp100s are hexamers with a central cavity in which misfolded polypeptides are being unfolded; and the Hsp70s are active as monomers and dimers without a central cavity. Yet, they all use the energy of ATP hydrolysis to extend and partially unfold misfolded structures into transiently bound protein aggregates, thereby providing unfolded segments a renewed chance to spontaneously refold into their native conformation, even under non-equilibrium conditions that are inauspicious for the native state (Sharma et al., 2011; Goloubinoff et al., 2018) (Figure 2). But why has evolution favored the appearance of several different families of ATP-fueled protein unfolding chaperones, which are structurally and mechanistically so different?

### Misfolded Proteins Are Structurally and Functionally Diverse

**FIGURE 3:**
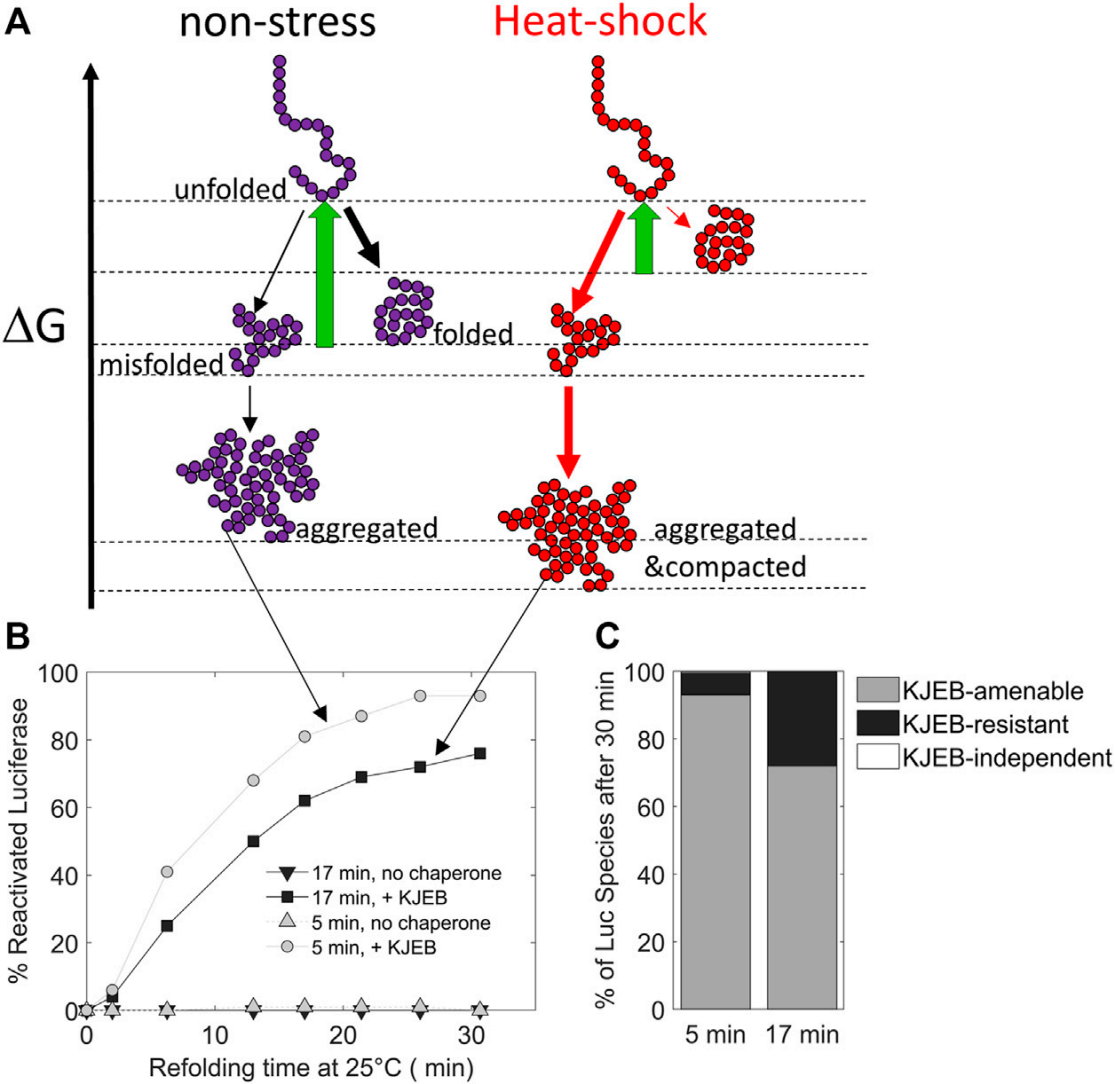
The duration of the heat-stress may change the position of protein conformers on a free energy scale. **(A)** free-energy diagram showing the various folding states of a polypeptide under non-stress (left) and heat-shock conditions (right, red). **(B)** time-dependent refolding of heat pre-denatured firefly luciferase (gray curves: 5 min denaturation at 45°C, black curves: 17 min at 45°C) in the presence or absence of DnaK, DnaJ, GrpE and ClpB (KJEB) at 25°C and ATP. **(C)** Deduced fraction of chaperone-amenable, chaperone-resistant and chaperone-independent luciferase species, determined after 30 min of refolding with KJEB at 25°C and ATP. Data from Goloubinoff (unpublished), based on similar experiments from (Diamant et al., 2000) and (Sharma et al., 2011).

Due to the structural diversity and complexity of the proteome, misfolded proteins can adopt a wide spectrum of non-native conformations, and depending on the nature and duration of stresses, a single native polypeptide may adopt a large array of misfolded and aggregated species with different properties (Figure 3).

Therefore, a single chaperone might not be able to process with the same efficiency all the possible different substrates. Instead, individual chaperone systems may preferentially bind to, and apply forceful unfolding onto, different kinds of misfolded intermediates, with various efficacies (Mapa et al., 2012; Tiwari et al., 2013). Thus, different chaperone systems expressed in the same cellular compartment can function synergistically, sequentially processing misfolded substrates, each by their corresponding most efficient chaperone. This is exemplified by the Hsp60 and Hsp70 systems from bacteria: while Hsp70 (DnaK) can efficiently disentangle highly compact preheated MDH aggregates into small, partially misfolded intermediates, but remains rather ineffective at converting them into native proteins, Hsp60 is totally unable to disaggregate stable MDH aggregates, but is highly effective at converting monomeric misfolded MDH intermediates into native proteins. Therefore, when both Hsp60 and Hsp70 are together, the conversion of MDH aggregates into native is most effective and rapid (Veinger et al., 1998) (Figure 2B). Similarly, whereas Hsp70, but not Hsp90, can disaggregate and unfold misfolded species, Hsp90 but not Hsp70 can accelerate the maturation of stalled Hsp70-intermediates, into native proteins (Genest et al., 2011; Morán Luengo et al., 2018; Morán Luengo et al., 2019).

### Principal Components of the Proteolytic Network

**FIGURE 4:**
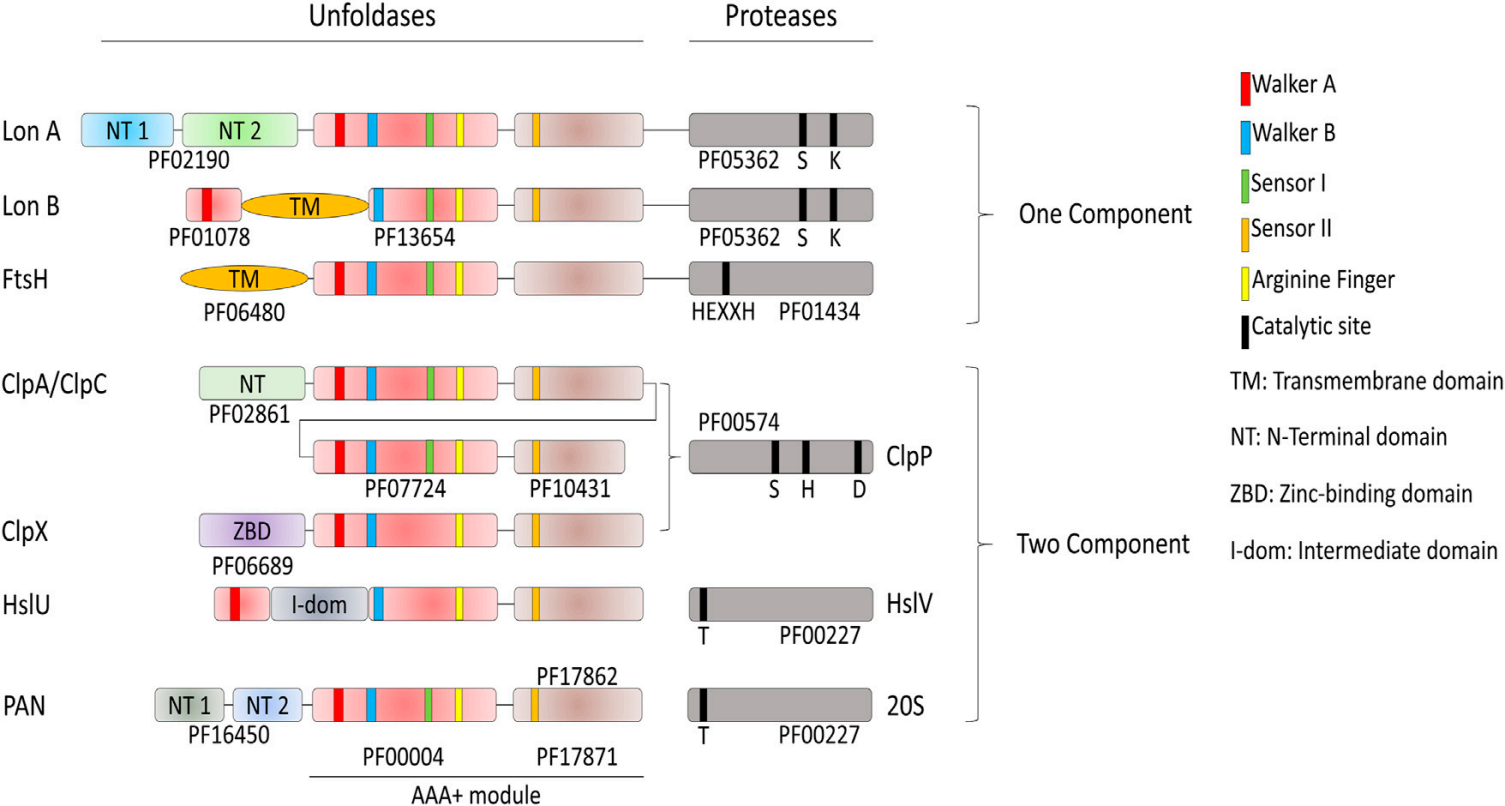
Domain organization of the AAA+ proteases of Bacteria and Archaea. Proteases have different catalytic sites; HslV and the archaeal 20S peptidase use an N-terminal threonine as the active-site nucleophile. Lon proteases have a lysine-serine dyad, ClpP uses a serine-histidine-arginine catalytic triad. FtsH is a Zn^2+^-dependent peptidase, with the specific HEXXH motif acting as zinc ligand. The AAA+ module is composed of the conserved PFAM domains PF00004 and PF17871 for every protease if not specified by another PFAM. ClpA and ClpC are composed of two AAA+ modules.

The *degrade-to-replace* section of the *E. coli* protein homeostasis network is composed of AAA+ proteases, namely FtsH, Lon, HslUV (also referred as ClpYQ), ClpXP and ClpAP, with the addition, in actinobacteria and Archaea of the eukaryotic-like MPA-20S proteasome (Neuwald et al., 1999; Iyer et al., 2004; Olivares et al., 2016) (Figure 4).

In contrast to the conserved chaperone families that vary extensively in structure and molecular mechanism, the conserved families of quality-control proteases share a common ancestor with the same structural features and a similar molecular mechanism to specifically degrade tagged or misfolded proteins: All are hollowed cylinders, with on their N-terminal side a hexameric ring of AAA+ domains, as in the case of Lon and FtsH, or a hexameric ring of independent AAA+ subunits gating a heptameric protease chamber, as in the case of ClpX, ClpA and HslU and PAN-20S (Figure 2). Proteases can specifically recognize and bind few specific alternatively folded, and in general misfolded protein substrates to be degraded, some in collaboration with N-terminal adaptors, such as ClpS in the case of the ClpAP protease (Alhuwaider and Dougan, 2017). Because of the high similarity of sequence and of oligomeric organization, the proteases likely use aa very similar mechanism of action: the energy of ATP hydrolysis is first harnessed to the forceful pulling, and thereby local unfolding, of stably misfolded or alternatively folded loops that are protruding from oligomers or aggregates. This is followed by the threading of the loops within the central cavity of the hexameric AAA+ cylinder, and their forceful extension (Deville et al., 2019), thereby activating and feeding an otherwise inaccessible catalytic proteolytic chamber, which is either found at the C-terminus (LonA and FtsH), or as a separate polypeptide (ClpAP and ClpXP systems, Figure 2). Thus, the participation of ATP-fueled unfoldases is a common feature to both the protein-repair and protein degrade-to-replace sides of the protein homeostasis network, in an attempt to unfold and spontaneously recover aggregated proteins into functional native proteins, or alternatively to unfold, thread and degrade them into peptides and free amino acids products of the peptidases, to be reused for the synthesis of new polypeptides.

### “Alter-Native States”: Chaperones and Proteases Recognize More Than Misfolded Proteins

**FIGURE 5:**
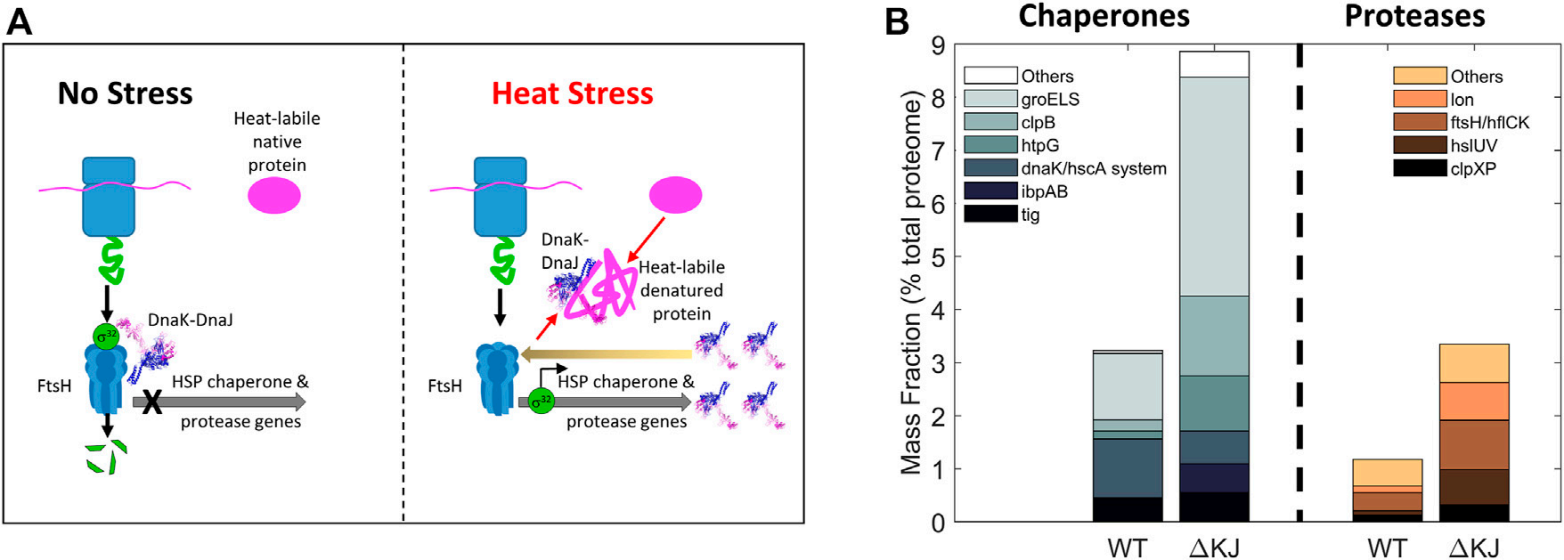
Collaboration between Hsp70 and a protease. **(A)** Bacterial mechanism for the heat-induced expression HSP-chaperones and proteases. **Left**: In the absence of stress, the transcription factor σ^32^ is bound by DnaK/DnaJ as is presented to the protease FtsH to be rapidly degraded, thereby preventing σ^32^-mediated transcription of heat-shock genes. **Right**: under heat stress, misfolded heat-labile proteins recruit DnaK/DnaJ (thick black arrows) and thus displace them from binding to σ^32^, allowing it to accumulate and resulting in the up-regulation of heat-shock proteins. The resulting excess of DnaK may then cause the degradation of σ^32^ and arrest excess HSP synthesis (back arrow). **(B)** Deletion of DnaK-DnaJ upregulates the steady state cellular levels of the chaperone-base repair and protease-based degradation machineries. Mass fraction (% of total proteome) of the chaperone and co-chaperones (left) and of proteases (right) in wild type and a delta DnaK-DnaJ *E. coli* strain. Protein quantities are shown as mass fractions (in % of the total proteome) of individual bacterial molecular chaperones and proteases (Data from (Fauvet et al., 2021)).

Diversification of biological processes occurs in different cellular environments, such as the cytosol where both chaperones and proteases are present and the endoplasmic reticulum where chaperones control the quality of the proteins to be secreted. It is thus not surprising that some physiological cellular pathways unrelated to protein misfolding have become “chaperone addicted” in the course of evolution and behave as chaperone-dependent substrates under particular conditions. In eukaryotes, this is the case of clathrin-cages that, following endocytosis, become specifically dismantled into their triskelia constituents by the cytosolic Hsc70 chaperones (Jiang et al., 2003; Sousa et al., 2016). Similarly, the protein IscU of the iron-sulfur cluster assembly pathway can assume two conformations whose populations are regulated by members of the Hsp70 family (Cupp-Vickery et al., 2004; Hoff et al., 2000). Thus, in the course of evolution, some proteins have evolved to use chaperones to switch between different “alter-native” and native conformations, none of which being per se, either structurally or functionally compromised. The bacterial heat shock transcription factor σ^32^ from *E. coli* is another example of such an “alternatively”-folded protein that is both a chaperone- and a protease-substrate. At non-heat shock temperatures, cellular σ^32^ levels are very low because it is constantly produced but it is also constantly unfolded by DnaK/DnaJ and delivered to the FtsH protease for degradation (Herman et al., 1995), resulting in a relatively low basal cellular expression level of the HSP genes (Taylor et al., 1984; Straus et al., 1987; Grossman et al., 1987). During heat shock, DnaJ and DnaK become recruited by labile heat-denaturing proteins, thereby reducing the amount of DnaK that can bind σ^32^ and present it to the protease FtsH for degradation. Consequently, σ^32^, which is less degraded by FtsH (Herman et al., 1995), binds more HSP promoters and activates the transcription of HSP genes (Figure 5). The consequent cellular accumulation of DnaK, DnaJ and FtsH in turn restores the degradation of excess σ^32^ and reduces the expression of HSPs in the long term (Straus et al., 1989). Here, the combined action of an unfolding chaperone and a protease is used to control their own expression. In eukaryotic cells, the major conserved orchestrator of the heat shock response, HSF1, functions in a similar manner to σ^32^. In unstressed cells, its “alternatively-folded” state is monomeric, maintained in this form in the cytosol by binding to multiple chaperones, notably HSP60s (Neef et al., 2014), HSP70s (Shi et al., 1998) and HSP90s (Ali et al., 1998). Upon stress, they release HSF1 upon to perform their stress-remediating activities. The released HSF1 is then able to reach its native trimeric form, that translocates to the nucleus to activate genomic heat shock elements (Trinklein et al., 2004).

In eukaryotic cells, a substantial fraction of the substrates that are being hydrolyzed by the ATP-dependent Ubiquitin-Proteasome system (UPS) (Glickman and Ciechanover, 2002) consists of natively folded functional proteins, which do not present large hydrophobic patches on their surfaces through which they could interact with chaperone and protease unfoldases. These short and medium half-life proteins, like transcription factors, cyclins or second intracellular messengers, have long been considered to be the main substrates of the proteasomal degradation system, rather than long half-life and damaged proteins (Lecker et al., 2006). To cope with that, a system had to evolve to recognize with extremely high efficiency and selectivity, the few proteins that must be rapidly degraded (Prakash et al., 2004; Yu et al., 2016). Therefore, this system labels them with a degradation tag (polyubiquitin) that can associate with specific receptors on the 19S cap of the regulatory particles gating the access to the 20S core particle in the 26S Proteasome (Marshall and Vierstra, 2019; Martinez-Fonts et al., 2020). Specifically, three proteasome subunits, Rpn1, Rpn10, and Rpn13 recognize the polyubiquitin chains. In a recent work, Cresti and colleagues demonstrated that the conformational changes of the 26S Proteasome control the unfolding capacity (Cresti et al., 2021). This selectivity has a considerable energy cost (synthesis of ubiquitin molecules, and then of the isopeptide linkage (Ciechanover et al., 1980; Wilkinson et al., 1980; Hershko et al., 1983)) and the degradation of these folded and functioning proteins has a very high energy cost, both for unfolding and for ubiquitin binding. Yet this is necessary as prolonged accumulation of these proteins would cause perturbation of cellular homeostasis, rendering it incompatible with life.

What are the thermodynamic implications of the protein repair and degrade-to-replace networks? With an amino acid pool (free or inserted into polypeptides), which is roughly constant (Hans et al., 2003; Roe et al., 1998)), the degrade-to-replace machinery is necessary to adapt the concentrations of different proteins to internal and external cues. Yet, whereas under stationary conditions errors of folding may be rare, under exponential growth, more spontaneous protein misfolding and aggregation may occur, and higher concentrations of chaperones and proteases may be needed to cope with this rare condition in nature. Purely relying on proteases to disassemble wrongly folded polypeptides would be both risky and energetically costly. On the one hand, some misfolded conformations, and even more so aggregates, might resist the unfolding necessary for threading through the AAA+ gate to be able to reach the proteolytic chamber, leading to progressive accumulation of waste in the cell that can be cytotoxic. For this case, eukaryotes have developed chaperone-mediated autophagy and micro-autophagy (Dong et al., 2020; Schuck, 2020). On the other hand, synthesizing, degrading and re-synthesizing proteins that have misfolded, so to maintain their native concentration at the correct cellular level, requires energy from ATP and GTP hydrolysis by several components of the degrade-to-replace pathway: the AAA+ that gate the proteases hydrolyze ATP to unfold their substrates; the aminoacyl-tRNA transferases that hydrolyze ATP to load amino acids onto their specific tRNAs and form high energy containing aminoacyl-tRNAs that bring the amino acids one-by-one to the ribosome for spontaneous oligomerization; the translation initiation factors that consume GTP molecules. Taking furthermore into account the ATP molecules that are necessary for the ubiquitination of proteasome substrates, and for pupylation in some bacteria (Pearce et al., 2008), degrading and re-synthesizing a new protein can cost a great number of nucleotide molecules (considering the energy from GTP hydrolysis similar to the one from ATP) that is at least equal to, but likely significantly greater than the free energy contained in the peptide bonds. As such, it is estimated that *de* novo protein synthesis costs about 4.2 ATP equivalents per residue (including the energy costs of mRNA synthesis) (Kaleta et al., 2013) (Figure 1). In contrast the hydrolysis of as little as five ATPs has been shown to suffice for one bacterial Hsp70 chaperone to convert one stably misfolded luciferase polypeptide containing 564 peptide bonds, into a stable, natively refolded enzyme (Sharma et al., 2010). Thus, the targeted degradation and replacement of damaged proteins necessitates at least two orders of magnitude more ATP than repairing damaged protein conformations by unfolding chaperones. We can thus expect that protein degradation should occur at a rate slow enough to allow a few tens of ATP-fueled chaperone protein-repair cycles per misfolded protein, but not significantly longer, to avoid wasting energy by trying to repair unrecoverable polypeptides.

Following proteolysis, exercising or starving animals may further degrade free amino acids into water, CO_2_, urea and/or ammonia, rather than recycling them into new proteins, thereby utilizing them as energy sources via oxidation (Figure 1) (Jungas et al., 1992). Extending the proposed view, these simpler compounds represent states of even lower free-energy for the atoms that were part of the amino acids. Therefore, an external energy source is needed to bring them back together into high-energy organic molecules, from which a series of mostly spontaneous metabolic reactions can replenish cells with the needed amino acids: photosynthesis was thus placed on the general free energy landscape of proteins (Figure 1). Photosynthetic organisms, such as cyanobacteria, can directly use the energy from the Sun and produce reduced amino acids from water, CO_2_ and N_2_, to be used by them and other organisms in the food chain, for the synthesis of their own polypeptides. Other non-photosynthetic organisms must rely on high-energy molecules, which they can take from the environment as a source of energy, to drive their metabolic processes that lead to the synthesis of amino-acids and nucleotides.

Protein conformational homeostasis in the cell is thus a series of nested cycles, comprising both spontaneous exergonic and energy-consuming endergonic processes (Figure 1). The degrade-to-replace cycle requires amino acids to be “pumped” up into higher-energy aminoacyl-tRNAs, and then polymerized into unfolded polypeptides in the ribosomes. From there, the inevitable tendency of all systems to progress toward their free-energy minimum leads to protein folding, but also, on a different path, to protein misfolding and aggregation, and to degradation of wrongly folded proteins, thus back to free amino acids, whose pool can be further reduced by amino acid degradation. In this case, re-synthesis of amino acids would be part of the degrade-to-replace cycle. This cycle is extremely energy-consuming, with a number of hydrolyzed nucleotides growing at least linearly with the length of the proteins to be degraded and replaced. The protein-repair cycle is nested within the degrade-to-replace one, and its role is, from an energetic perspective, to reduce the necessity of obligatory degradation, by rescuing misfolded and aggregated proteins and giving them an opportunity to refold properly. The unfolding action of chaperones is likely related to the intrinsic stability of individual misfolded domains, and it is thus able to produce an unfolded polypeptide at a lesser energy cost than its *de novo* synthesis. The repair machinery thus represents a more parsimonious approach for protein conformational homeostasis. Nonetheless, *degrade-to-replace* may become unavoidable and ultimately more energetically advantageous when chemical modifications of proteins, such as glycation, oxidation, and unwanted partial proteolysis could prevent efficient chaperone-driven structural repair, leading to many wasteful ATPase cycles (Chondrogianni et al., 2014). In Cuba under embargo, 60 years of increasingly expensive iterative cycles of repair of old American cars have likely cost their owners much more than if they would have been given the possibility to replace them, even by pricy new cars (which in their case were unavailable).

## Conclusion

The thermodynamic dilemma of the proteostasis machinery of all organisms is whether it is more energetically convenient to repair or to replace stress-damaged proteins. The protein homeostasis network is composed of abundant “holding” and ATP-fueled unfolding chaperones that can respectively prevent aggregation and actively repair misfolded proteins into native ones, at a relatively low ATP cost. Yet, when proteins are irreversibly damaged, either chemically or structurally, ineffective attempts of repair would waste ATP, which is to be avoided. ATP-fueled unfolding proteases, although unlikely to be activated by chaperones, may yet specifically recognize the chaperone-stalled, resistant misfolded species, bind them, unfold and funnel them into the proteolysis chambers of the proteases for degradation, while maintaining untouched a large excess of surrounding functional proteins which are native. Although degradation is to be followed by costly ATP and GTP consuming re-synthesis, proteins are like used cars: There always comes a point where the cost of cumulative repairs exceeds that of buying a new car.
